# Supplementary material for: Supermoms—Tired, admired, or inspired? Decoding the impact of supermom beliefs: A study on Indian employed mothers
Source: PLoS One. 2025 Apr 23;20(4):e0321665. doi: 10.1371/journal.pone.0321665 (PMC12017522; doi:10.1371/journal.pone.0321665)
Supplement: Attachment — Submitted filename: Response to the Reviewers.pdf [file pone.0321665.s002.pdf]

To,

The Editor and Reviewers,

PLOS ONE Date: February 9, 2025

Subject: Addressing Editor/Reviewer Comments for PONE-D-24-24738

Dear Editor and Reviewers,

We appreciate the opportunity to revise and resubmit our manuscript titled "Supermoms - Tired, admired, or inspired? Decoding the impact of supermom beliefs: A study on Indian employed mothers." We are grateful to the reviewers for their insightful comments and suggestions, which have helped us improve the quality of our work. Below, we provide a detailed response to each reviewer's comments, outlining the revisions made to the manuscript. The responses to the reviewers' comments and all changes in the revised manuscript with track changes are highlighted in blue.

Thank you,

Kind regards,

Authors of PONE-D-24-24738

PONE-D-24-24738: Supermoms - Tired, admired, or inspired? Decoding the impact of supermom beliefs: A study on Indian employed mothers

**Email received regarding the revision and resubmission:**

Thank you for submitting your manuscript to PLOS ONE. After careful consideration, we feel that it has merit but does not fully meet PLOS ONE's publication criteria as it currently stands. Therefore, we invite you to submit a revised version of the manuscript that addresses the points raised during the review process.

Following are few observations, which require considerate response:

- Literature review should include a section on theoretical rationale to justify the objectives and hypotheses formulated. We have included this on Page 8 and 9 under the title Theoretical Rationale.

- Hypothesis 1 is not according to APA style. We have changed this and rechecked other hypotheses for the same reason.

- 'Supermom Belief' was measured qualitatively and then grouped into two themes as 'beneficial' or 'detrimental' whereas, path analysis only refers to the beneficial category. It is not clear whether this type of data justifies the use of path analysis. For quantitative analysis, we have used a dichotomous ordinal variable to capture 'Supermom' beliefs. We have coded them as 1 and 0 to denote 'beneficial' and 'detrimental' beliefs respectively. Literature (Muthén, 1984; Kuha and Goldthorpe, 2010; Li, 2021) suggests that this can be used for path analysis if the continuous variables in the model have a normal distribution. We have confirmed the normal distribution and then proceeded with path analysis. The section on 'Quantitative Method' on Pages 21 and 22 explains this in further detail.

  Similarly, Pearson correlation has been applied to find the association between SMB and other variables. Thank you for pointing this out. We have replaced Pearson with Spearman's Correlation for better suitability (Page 23 and in Table 3, Page 24).

- The whole article needs to be reviewed for grammatical mistakes. We have fixed the problem and thoroughly checked our text for grammatical inconsistencies with Grammarly.

Please submit your revised manuscript by Feb 13 2025 11:59PM. If you will need more time than this to complete your revisions, please reply to this message or contact the journal office at plosone@plos.org. When you're ready to submit your revision, log on to https://www.editorialmanager.com/pone/ and select the 'Submissions Needing Revision' folder to locate your manuscript file.

Please include the following items when submitting your revised manuscript:

- A rebuttal letter that responds to each point raised by the academic editor and reviewer(s). You should upload this letter as a separate file labeled 'Response to Reviewers'. Included
- A marked-up copy of your manuscript that highlights changes made to the original version. You should upload this as a separate file labeled 'Revised Manuscript with Track Changes'. Included
- An unmarked version of your revised paper without tracked changes. You should upload this as a separate file labeled 'Manuscript'. Included

If you would like to make changes to your financial disclosure, please include your updated statement in your cover letter. Guidelines for resubmitting your figure files are available below the reviewer comments at the end of this letter.

If applicable, we recommend that you deposit your laboratory protocols in protocols.io to enhance the reproducibility of your results. Protocols.io assigns your protocol its own identifier (DOI) so that it can be cited independently in the future. For instructions see: https://journals.plos.org/plosone/s/submission-guidelines#loc-laboratory-protocols. Additionally, PLOS ONE offers an option for publishing peer-reviewed Lab Protocol articles, which describe protocols hosted on protocols.io. Read more information on sharing protocols at https://plos.org/protocols?utm_medium=editorial-email&utm_source=authorletters&utm_campaign=protocols.

We look forward to receiving your revised manuscript.

Kind regards,

Shazia Khalid, PhD
Academic Editor
PLOS ONE

----------------------------------------------------------------------------------------------------------------

Journal requirements:

1. When submitting your revision, we need you to address these additional requirements.

Please ensure that your manuscript meets PLOS ONE's style requirements, including those for file naming. The PLOS ONE style templates can be found at

https://journals.plos.org/plosone/s/file?id=wjVg/PLOSOne_formatting_sample_main_body.pdf
and

https://journals.plos.org/plosone/s/file?id=ba62/PLOSOne_formatting_sample_title_authors_affiliations.pdf

We have followed the PLOS One template available at https://journals.plos.org/plosone/s/file?id=wjVg/PLOSOne_formatting_sample_main_body.pdf

2. We note that you have indicated that there are restrictions to data sharing for this study. PLOS only allows data to be available upon request if there are legal or ethical restrictions on sharing data publicly. For more information on unacceptable data access restrictions, please see http://journals.plos.org/plosone/s/data-availability#loc-unacceptable-data-access-restrictions.

We have fixed this, by publishing the data on 10th December 2024 on Mendeley.com, where the data is freely and publicly available. The URL to access the data: Shah, Dr Shalaka (2024), "Data of Indian Mothers", Mendeley Data, V1, doi: 10.17632/2jtx5864yx.1

Before we proceed with your manuscript, please address the following prompts:

a) If there are ethical or legal restrictions on sharing a de-identified data set, please explain them in detail (e.g., data contain potentially identifying or sensitive patient information, data are owned by a third-party organization, etc.) and who has imposed them (e.g., a Research Ethics Committee or Institutional Review Board, etc.). Please also provide contact information for a data access committee, ethics committee, or other institutional body to which data requests may be sent.

There are no ethical or legal restrictions on sharing the data publicly. We had mentioned initially that the data would be available upon request, but considering this is a prerequisite for publishing for PLOS ONE, we decided to make the data available publicly.

b) If there are no restrictions, please upload the minimal anonymized data set necessary to replicate your study findings to a stable, public repository and provide us with the relevant URLs, DOIs, or accession numbers. For a list of recommended repositories, please see

https://journals.plos.org/plosone/s/recommended-repositories. You also have the option of uploading the data as Supporting Information files, but we would recommend depositing data directly to a data repository if possible.

Thank you for this suggestion. We have done exactly what you have suggested. We published the data on 10th December 2024 on Mendeley.com, where the data is freely and publicly available. The URL to access the data is Shah, Dr Shalaka (2024), "Data of Indian Mothers", Mendeley Data, V1, doi: 10.17632/2jtx5864yx.1

We will update your Data Availability statement on your behalf to reflect the information you provide.

Thank you for updating the Data Availability Statement for us!

3. In the online submission form, you indicated that [Data cannot be shared publicly as it includes their email addresses. But Data can be made available from the Corresponding Author / Ethics Committee (contact via shalaka.shah@flame.edu.in) for researchers who meet the criteria for access to confidential data.].

All PLOS journals now require all data underlying the findings described in their manuscript to be freely available to other researchers, either 1. In a public repository, 2. Within the manuscript itself, or 3. Uploaded as supplementary information.

This policy applies to all data except where public deposition would breach compliance with the protocol approved by your research ethics board. If your data cannot be made publicly available for ethical or legal reasons (e.g., public availability would compromise patient privacy), please explain your reasons on resubmission and your exemption request will be escalated for approval.

We have addressed this by publishing the data. Kindly see the response to point no. 2 above.

4. Please review your reference list to ensure that it is complete and correct. If you have cited papers that have been retracted, please include the rationale for doing so in the manuscript text, or remove these references and replace them with relevant current references. Any changes to the reference list should be mentioned in the rebuttal letter that accompanies your revised manuscript. If you need to cite a retracted article, indicate the article's retracted status in the References list and also include a citation and full reference for the retraction notice.

We have checked the reference list, and the list is updated with the additions we have made based on the valuable feedback from the reviewers. We checked and to the best of our knowledge, we haven't cited articles that have been retracted.

----------------------------------------------------------------------------------------------------

Reviewers' comments:

Reviewer's Responses to Questions

**Comments to the Author**

1. Is the manuscript technically sound, and do the data support the conclusions?

The manuscript must describe a technically sound piece of scientific research with data that supports the conclusions. Experiments must have been conducted rigorously, with appropriate

controls, replication, and sample sizes. The conclusions must be drawn appropriately based on the data presented.

Reviewer #1: Yes

Reviewer #2: Partly

Thank you for this feedback.

---

2. Has the statistical analysis been performed appropriately and rigorously?

Reviewer #1: Yes

Reviewer #2: Yes

Thank you very much for this feedback.

---

3. Have the authors made all data underlying the findings in their manuscript fully available?

The PLOS Data policy requires authors to make all data underlying the findings described in their manuscript fully available without restriction, with rare exception (please refer to the Data Availability Statement in the manuscript PDF file). The data should be provided as part of the manuscript or its supporting information, or deposited to a public repository. For example, in addition to summary statistics, the data points behind means, medians and variance measures should be available. If there are restrictions on publicly sharing data—e.g. participant privacy or use of data from a third party—those must be specified.

Reviewer #1: No

Reviewer #2: No

We have made the data available publicly. The DOI to access the data is 10.17632/2jtx5864yx.1

---

4. Is the manuscript presented in an intelligible fashion and written in standard English?

PLOS ONE does not copyedit accepted manuscripts, so the language in submitted articles must be clear, correct, and unambiguous. Any typographical or grammatical errors should be corrected at revision, so please note any specific errors here.

Reviewer #1: Yes

Reviewer #2: No

Thank you for the feedback. We have rechecked the whole document for grammatical errors and proofread it for clarity and correctness.

---

5. Review Comments to the Author

Please use the space provided to explain your answers to the questions above. You may also include additional comments for the author, including concerns about dual publication, research ethics, or publication ethics. (Please upload your review as an attachment if it exceeds 20,000 characters)

**Reviewer #1:** The article provides a valuable exploration of how societal ideals around motherhood affect the self-efficacy, work engagement, and psychological well-being of Indian employed mothers. Overall the study gave a comprehensive picture of the title but here are some recommendations that can improve the quality of the study.

We are grateful for your insightful comments and suggestions, which have helped us improve the quality of our work. Below, we provide our responses highlighted in blue. All the changes in the manuscript have also been highlighted in blue.

1. The most important part that requires attention is the methodology of Qualitative part of study. Author must clearly state in detail about the methodological framework used to develop open ended questions.

Thank you for pointing out this gap. We have reworked the qualitative method section and included symbolic interactionism as one of the most suitable qualitative frameworks. These additions are made on Pages 16, 17, 18 and 19. We have tied up this framework and its connection to the study variables in the Introduction section on Pages 2 and 3.

2. The term 'supermom' is central to the study, but it lacks a precise and consistent definition across the article. While the qualitative responses attempt to capture different viewpoints, the notion remains somewhat vague. A clearer operational definition of what qualifies as 'supermom' behavior or expectations would help in interpreting the results.

Thank you for this feedback. We have included a holistic definition of supermom on Page 3 (Introduction) and Page 18 (end of the Qualitative Data Analysis & Results section, line no. 379-382).

3. considering and highlighting limitations and future recommendations would be a good addition to the article. The limitations in sampling, cultural diversity, and studying depth of negative consequences suggest room for improvement (Longitudinal study). Recommending these points in future research would enhance the scope and impact of the findings.

Thanks for these valuable suggestions. We have reworked the Limitations and Future Recommendations section on Pages 33 and 34 as per these suggestions.

----------------------------------------------------------------------------------------------------

**Reviewer #2:** This study provides valuable insights into the psychological challenges faced by employed mothers in India, shedding light on the impact of "supermom" beliefs. The research is strong in many areas. However, there are gaps By addressing the following aspects, the authors could further strengthen their work.

We are grateful for your insightful comments and suggestions, which have helped us improve the quality of our work. Below, we provide our responses highlighted in blue. All the changes in the manuscript have also been highlighted in blue.

1. The entire document requires careful editing to correct grammatical errors and restructure overly long sentences for improved readability and flow.

Thank you for the feedback. We have rechecked the whole document for grammatical errors and proofread it for clarity, correctness, and flow.

2. Focus on the most critical aspects of the introduction, eliminating repetitive details to ensure a concise and engaging narrative.

This feedback helped us reconsider our narrative. We have reworked the introduction, including a clear definition of the supermom notion, added the perspective of societal myths and ideals and how they shape the notion of supermom, and rechecked the flow of various aspects we present. All changes can be found on Pages 3 and 4 and are marked in blue.

3. The structural model is currently discussed in three different sections of the paper—under the "Measures" section, before hypothesis testing, and after the results. To improve clarity and

coherence, it would be more effective to integrate these discussions into a single, unified section, ensuring a smooth flow of information and avoiding redundancy.

Thank you for pointing this out. We have combined these discussions under the broad heading of ‘Quantitative method section’ (Pages 21 to 25)

4. The authors have taken an important step by conducting EFA and reporting reliability, the absence of methodological details, validity metrics, and contextual interpretations weakens the transparency and robustness of the findings. Addressing these gaps will enhance the credibility and impact of the analysis.

Thank you for your valuable input. We have incorporated the methodological details in the first paragraph on Page 21 (line no. 413 to 423) and validity metrics in the second paragraph of ‘Quantitative Data Analysis & Results’ on Page 22, line no. 443 to 467.

We have included contextual interpretations in the last paragraph of the ‘Discussion’ section on Page 32, line no. 635 to 641.

5. Provide additional details about the path analysis process, particularly addressing the use of the "Supermom Beliefs as Beneficial" variable, which was nominal and not appropriate for Pearson correlation.

Thank you for your advice. We have now clarified that "Supermom Beliefs as Beneficial" is a dichotomous ordinal variable to capture ‘Supermom’ beliefs. We have coded them as 1 and 0 to denote ‘beneficial’ and ‘detrimental’ beliefs respectively. Literature (Muthén, 1984; Kuha and Goldthorpe, 2010; Li, 2021) suggests that this can be used for path analysis if the continuous variables in the model have a normal distribution.

We have confirmed the normal distribution and then proceeded with path analysis. The section on ‘Quantitative Method’ on Pages 21 explains in further detail.

We have further replaced Pearson with Spearman’s Correlation for its better suitability (Table 3, Page 23).

6. Include bootstrapped estimates of indirect effects and confidence intervals to substantiate claims regarding mediation.

Thank you for the suggestion. We have included bootstrapped estimates of indirect effects and confidence intervals at the end of Hypotheses Testing (Table 6, Pages 27).

7. The significant chi-square statistic suggests a potential misfit between the model and the data. While this is common in large samples, the authors should explicitly discuss the limitations of the chi-square test and justify reliance on alternative fit indices.

Thank you for your feedback. We have explicitly discussed the limitations of the chi-square test and justified reliance on alternative fit indices, which we have explained in detail, towards the end of the 'Quantitative Data Analysis & Results' section before Hypotheses Testing (Pages 24 and 25, line no 480 to 498)

8. Tie the findings to established psychological theories, such as self-efficacy theory or theories of work engagement, to provide a more nuanced explanation of the results.

This has been addressed in the Results section by including Role Theory to understand the qualitative analysis better on Page 19, line no. 393-399.

We have added more theories in the discussion section on Pages 29 and 30. We have included Self-efficacy Theory, Conservation of resources theory, Broaden and Build theory in Theoretical rationale section line no. 171 to 182, and Role Enhancement Theory line no. 588-591

9. Discuss how these findings can inform interventions for employed mothers, including workplace policies and mental health initiatives.

This has been added in the practical implications section on Pages 32 and 33

10. Consider potential confounders or alternative explanations for the observed relationships, and explicitly acknowledge the limitations of the study design.

We have reworked the limitations and future research sections on Pages 33 and 34 to address this comment.

---

6. PLOS authors have the option to publish the peer review history of their article (what does this mean?). If published, this will include your full peer review and any attached files.

If you choose "no", your identity will remain anonymous but your review may still be made public.

**Do you want your identity to be public for this peer review?** For information about this choice, including consent withdrawal, please see our Privacy Policy.

Reviewer #1: No

Reviewer #2: No
